# Effect of an intensive nutrition intervention of a high protein and low glycemic-index diet on weight of kidney transplant recipients: study protocol for a randomized clinical trial

**DOI:** 10.1186/s13063-017-2158-2

**Published:** 2017-09-06

**Authors:** Elis Forcellini Pedrollo, Bruna Bellincanta Nicoletto, Larissa Salomoni Carpes, Júlia de Melo Cardoso de Freitas, Julia Roberta Buboltz, Cristina Carra Forte, Andrea Carla Bauer, Roberto Ceratti Manfro, Gabriela Corrêa Souza, Cristiane Bauermann Leitão

**Affiliations:** 1 Post Graduate Program in Health Sciences, Endocrinology, Universidade Federal do Rio Grande do Sul, Rua Ramiro Barcelos, 2400, 90035-003 Porto Alegre, RS Brazil; 2 Nutrition Course, Knowledge Area of Life Sciences, Universidade de Caxias do Sul, Caxias do Sul, RS Brazil; 3 Nutrition Graduate Course, School of Medicine, Federal University of Rio Grande do Sul, Porto Alegre, RS Brazil; 4 Department of Internal Medicine, School of Medicine, Universidade Federal do Rio Grande do Sul, Rua Ramiro Barcelos, 2400, 90035-003 Porto Alegre, RS Brazil; 5 Post Graduate Program in Medicine: Medical Sciences, Universidade Federal do Rio Grande do Sul, Rua Ramiro Barcelos, 2400, 90035-003 Porto Alegre, RS Brazil; 6 Department of Nutrition, School of Medicine, Universidade Federal do Rio Grande do Sul, Porto Alegre, RS Brazil; 7 Food and Nutrition Research Center, Hospital de Clínicas de Porto Alegre (HCPA-UFRGS), Porto Alegre, RS Brazil

**Keywords:** Kidney transplantation, Nutrition intervention, High protein diet, Low glycemic-index diet, Weight

## Abstract

**Background:**

Excessive weight gain is commonly observed within the first year after kidney transplantation and is associated with negative outcomes, such as graft loss and cardiovascular events. The purpose of this study is to evaluate the effect of a high protein and low glycemic-index diet on preventing weight gain after kidney transplantation.

**Methods:**

We designed a prospective, single-center, open-label, randomized controlled study to compare the efficacy of a high protein (1.3–1.4 g/kg/day) and low-glycemic index diet versus a conventional diet (0.8–1.0 g/kg/day of protein) on preventing weight gain after kidney transplantation. A total of 120 eligible patients 2 months after transplantation will be recruited. Patients with an estimated glomerular filtration rate through the modification of diet of renal disease (MDRD) formula < 30 mL/min/1.73 m^2^ or urinary albumin excretion > 300 mg/24 h will be excluded. Patients’ diets will be allocated through simple sequential randomization. Patients will be followed-up for 12 months with nine clinic appointments with a dietitian and the evaluations will include nutritional assessment (anthropometrics, body composition, and resting metabolic rate) and laboratory tests. The primary outcome is weight maintenance or body weight gain under 5% after 12 months. Secondary outcomes include body composition, resting metabolic rate, satiety sensation, kidney function, and other metabolic parameters.

**Discussion:**

Diets with higher protein content and lower glycemic index may lead to weight loss because of higher satiety sensation. However, there is a concern about the association of high protein intake and kidney damage. Nevertheless, there is little evidence on the impact of high protein intake on long-term kidney function outcome. Therefore, we designed a study to test if a high protein diet with low-glycemic index will be an effective and safe nutritional intervention to prevent weight gain in kidney transplant patients.

**Trial Registration:**

ClinicalTrials.gov identifier, NCT02883777. Registered on 3 August 2016.

**Electronic supplementary material:**

The online version of this article (doi:10.1186/s13063-017-2158-2) contains supplementary material, which is available to authorized users.

## Background

Weight gain after kidney transplantation is very often observed and it has been reported to be between 10% and 35%, mainly during the first year after transplant [1–4]. Post-transplant overweight and obesity may lead to negative post-transplant outcomes, such as graft loss and cardiovascular events [5, 6]. In addition, weight gain during the first year post-transplantation appears to be a risk factor for the development of new-onset diabetes and metabolic syndrome [7–9]. The main factors implied in the weight gain in this population are the immunosuppressive regimen, the cessation of dietary restrictions associated with dialysis, consequent appetite restoration, and improvements in quality of life [10, 11].

Data on nutritional management to prevent weight gain after transplantation is scarce [12–17]. Moreover, the evidence assessing protein requirements in kidney transplant patients is also limited [15, 18]. High protein intake in the early period post-transplant is recommended to match protein catabolism, but there is no evidence available regarding long-term protein requirements of stable renal-transplanted recipients [18].

A high-protein diet is known to be effective for body weight loss and subsequent weight maintenance in the general population [19–22]. Protein generally exerts a better satiety effect than carbohydrates and lipids [23–25]. During the process of weight loss, a high-protein diet preserves lean body tissue, which is the major determinant of resting and 24-h energy expenditure, which, in turn, prevents a greater reduction in energy expenditure [23] usually observed in individuals undergoing a weight reduction program. Besides, it is well known that a diet with a low glycemic index (GI) is determinant of postprandial metabolic responses to food intake and also may have beneficial effects on body weight and body composition [26–28]. Larsen et al. [29] have shown that a dietary plan with moderately high protein associated with a slightly reduced GI leads to weight loss maintenance in overweight adults who had lost at least 8% of body weight. In this context, we designed a randomized clinical trial in order to evaluate the effect of a high protein and low GI diet in preventing weight gain after kidney transplantation.

## Methods/design

### Study design and centers

**Fig. 1:**
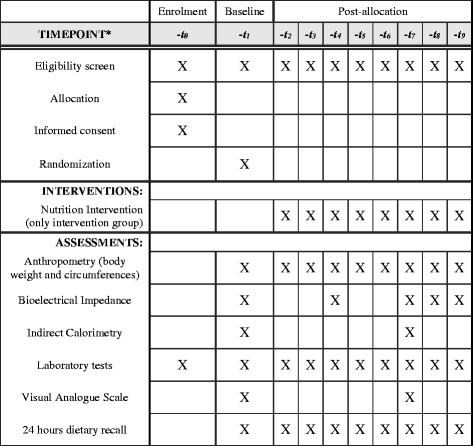
SPIRIT diagram. *Timepoint of the protocol: -t0, enrolment; -t1, baseline; -t2, -t3, -t4, -t5, -t6, -t7, first semester monthly appointments; -t8, month 9; -t9, month 12

This is a prospective, single-center, open-label, randomized clinical trial that will include an interventional group (high protein and low GI diet) and a control group (usual diet) patients that will undergo kidney transplant at Hospital de Clínicas de Porto Alegre, Brazil. The present protocol was written in accordance with Standard Protocol Items: Recommendations for Interventional Trials (SPIRIT) guideline, completing the SPIRIT checklist, and constructing a flow diagram in order to optimize the quality of reporting [30] (Fig. 1 and Additional file 1).

### Inclusion and exclusion criteria

The study will include kidney transplant recipients who agree to participate in the study protocol and provide written informed consent. The exclusion criteria will be the following: patients younger than 18 years old, prior transplant, multiple organ transplant, type 1 diabetes mellitus, current cancer, women in pregnancy or lactation period, recipients of a kidney from living donors, patients with urinary albumin excretion > 300 mg/24 h or estimated glomerular filtration rate through the modification of diet of renal disease (MDRD) formula < 30 mL/min/1.73 m^2^, and/or anticipated difficulty of adherence (for example, due to any kind of cognitive deficits or dementia).

### Sample size

Sample size calculations were carried out in WINPEPI 11.20 (Brixton Health, Israel) based on data from Souza et al. [31]. To find a difference of 5% in body weight between groups 1 year after the transplant, considering a standard deviation of 8.8%, a significance level of α ≤ 0.05 and a statistical power of 80%, the minimum sample size will be 98 patients. Because of possible dropouts, we will include 120 patients (60 randomized to each group).

### Study intervention

Patients will be randomized to: 1) an intervention group who will receive a high-protein diet (1.3–1.4 g/kg/day) with low GI; and 2) a control group that will receive a conventional diet that provides approximately 0.8–1.0 g/kg of protein intake. All the patients will be followed for 12 months with nine clinic appointments made by a research dietitian. The evaluations will include nutritional assessment (anthropometrics, body composition, and resting metabolic rate) and laboratory tests.

### Randomization

The randomization will be performed through a simple sequential randomization plan generated online (using the randomization.com website [32]) by another researcher (CCF).

### Blinding

In this clinical trial blinding of patients and dietitians is not possible because of evident differences between the intervention and control group.

### Data collection and timeline

Follow-up evaluation and data collection will be undertaken over 2 years and 6 months at the Clinical Research Center of the Hospital de Clínicas de Porto Alegre, Brazil, by trial personnel. All research tests will be assessed on the same day as protocol laboratory tests.

### Adherence and acceptability

In order to assess diet compliance and safety issues, all participants will collect 24-h urine samples to measure albumin, protein, creatinine, and urea excretion every 3 months. During the first semester, the subjects will have a monthly nutritional visit and, during the second semester, patients will be seen at month 9 and month 12 after randomization.

### Study protocol

Kidney transplant recipients who meet the inclusion criteria and are eligible will be invited to participate in this study 2 months after the transplant surgery. Patients will be randomized to the intervention group or the control group. The intervention group will receive a high-protein (1.3–1.4 g/kg/day) and low GI diet (preference for foods with a glycemic index ≤ 55%, with a daily glycemic load of ≤ 80 g), and the control group will receive a conventional diet (0.8–1.0 g/kg/day of protein). The protein requirement will be re-evaluated 6 months after the baseline. For patients that have lost or gained more than 5% of body weight, the diet will be recalculated. The intervention and control groups will receive energy-matched diets.

Demographic and clinical data will be assessed at the first visit. Nutritional assessment will consist of: a) anthropometric measurements (body weight, height, calculated body mass index (BMI; kg/m^2^), and waist circumference measured midway between the lowest rib margin and the iliac crest, with flexible no-stretch fiberglass tape) performed at each of the nine visits; b) body fat mass (%) measured by bioelectrical impedance analyzer (In Body 230, GE Health Care) assessed every 3 months; and c) resting metabolic rate evaluated by indirect calorimetry (Meta Check 7100, Metabolic Rate Analysis System, KOOR) at baseline and 6 months. All the nutritional measurements will be performed with the patient fasting, wearing light clothing, without contact with metals, and without shoes.

Biochemical assessment will include serum and urine creatinine (assessed monthly), fasting glycemia, cholesterol, high-density lipoprotein (HDL) cholesterol, triglycerides, glycated hemoglobin, and uric acid (assessed every 3 months), high-sensitivity C-reactive protein (at baseline and 6 months), and 24-h urine test for albumin, protein, creatinine, and urea excretion (every 3 months).

The diet prescription will be calculated using nutritional software (Nutribase 2007 Clinical Manager software version 7.14). GI will be estimated as proposed by the Food and Agriculture Organization (FAO) [33] using the international table–United States Department of Agriculture (USDA) table [34], with glucose as the standard food [35] and considering a daily glycemic load of ≤ 80 g. This will be considered as 1.3–1.4 g/kg/day of protein. Energy intake will be assessed by a 24-h recall over nine visits by the research dietitian. Diet composition also will be analyzed using the nutritional table with the software (Nutribase 2007 Clinical Manager software version 7.14) at each visit.

Two months after transplantation, patients are invited to participate in the study protocol and are randomized to the intervention or control group. The intervention group receives the study diet and the control group receives the conventional diet. Both groups visit the center once a month for the first 6 months. After that, another two visits are scheduled at months 9 and 12. At each visit both groups are submitted to the research anthropometric tests and the 24-h recall diet is completed. Besides the prescription of the intervention diet after randomization, the research dietitian reinforces diet adherence at each visit, but only for the intervention group. The standard diet adherence reinforcements are more sporadic for the control group (three to four visits scheduled per year) with the standard dietitian from the hospital. Thus, the intervention group receives eight diet reinforcements and the control group receives three to four reinforcements during the study protocol.

Food intake and adherence to the prescribed diet will be assessed by 24-h recall. An experienced registered dietitian will implement the recall during a face-to-face interview. To assure accurate answers, a photographic album of food portions and household measures will increase the precision of the amount of food consumed. A total of nine records over 1 year will be available for each included patient. Furthermore, the study protocol also includes the collection of urinary urea excretion and this adds to the calculation for the protein equivalent of total nitrogen appearance (nPNA) as a measure of dietary protein intake adherence.

Satiety levels will be assessed twice through a visual analogue scale (VAS) of appetite [36]. This scale will be answered by each patient at home 2 h after three main meals (breakfast, lunch, and dinner) at baseline and 6 months later.

### Primary outcome

The primary outcome will be weight maintenance and weight gain under 5% of body weight.

### Secondary outcomes

The secondary outcomes will consist of: a) body composition assessed every 3 months using a bioelectrical impedance analyzer (patients with 12-h fasting); b) resting metabolic rate evaluated by indirect calorimetry at baseline and 6 months later (patients with 12-h fasting); c) satiety evaluated twice (at baseline and 6 months later) by VAS and answered 2 h after three main meals (breakfast, lunch, and dinner); d) kidney function assessed by serum creatinine through estimated glomerular filtration rate (MDRD formula) and 24-h urine test with albumin and protein (every 3 months); e) glycated hemoglobin evaluated every 3 months; f) lipid profile evaluated every 3 months by total cholesterol, HDL cholesterol, and triglycerides (with 12-h fasting) laboratory tests; and g) inflammation assessed through high-sensitivity C-reactive protein at baseline and 6 months later.

### Statistical analyses

Continuous variables with normal distribution will be expressed as mean ± standard deviation. The Shapiro-Wilk test will be used for normality assessment; asymmetrically distributed continuous variables will be expressed as median and interquartile range; and categorical variables will be expressed as absolute and relative frequencies. For between-group comparisons, the Student’s *t* test will be used for normally distributed variables, and the Mann–Whitney *U* test for asymmetrically distributed variables. A paired *t* test will be used for within-group analysis of body weight and body composition. The Chi-square or Fisher’s exact tests will be used to evaluate associations between categorical variables. The generalized estimating equations test with Bonferroni adjustment will be used for comparison between variables during the study period. The significance level will be set lower than 5%, and all data will be analyzed in SPSS 20.0 (SPSS Inc., Chicago, IL, USA).

## Discussion

To the best of our knowledge, this is the first randomized clinical trial that will evaluate the impact of a high protein and low GI diet on weight maintenance or weight gain lower than 5% of body weight after kidney transplantation. Furthermore, this study will evaluate other relevant parameters related to metabolic outcomes since we hypothesized that this dietetic intervention may be able to improve body composition, resting metabolic rate, satiety, inflammation, and lipid and glycemic profiles.

Importantly, there is still concern related to a high protein intake and kidney damage based on some previous studies that showed an association between a high protein intake and worsening of renal function [37–40]. However, data on the impact of protein intake on long-term outcome in kidney transplant recipients are scarce [40–43]. Bernardi et al. evaluated a low-protein, low-lipid, and low-sodium diet in a 12-year follow-up study and showed a protective effect for the kidney with this diet [40], but the interpretation of the results are limited and controversial. Van Den Berg et al. [41] studied the association between protein intake and blood pressure, proteinuria, and creatinine clearance in a cross-sectional study with 625 renal transplant recipients, and no deleterious effects of the diet were identified. Interestingly, in a cohort of 940 kidney transplant recipients, a higher protein intake was associated with protection against mortality and graft failure [42]. These results were confirmed in a more recent cohort of 604 kidney transplant recipients with 7 years of follow-up [43]. Said et al. have shown that a high protein intake was associated with improvements in muscle mass and with reduced risk of mortality and graft failure [43], suggesting that a relatively high protein intake may be beneficial to kidney transplant recipients.

Other studies evaluating the impact of dietary interventions in kidney transplant recipients show conflicting results, mainly limited by the study design [12–14]. Thus, due to lack of high-quality evidence data on this issue, there are no guidelines or recommendations for a specific nutritional intervention to manage weight gain and obesity after kidney transplantation [44–46].

Since it is not possible to blind participants and researchers involved in this study, there are possible risks of bias. In order to diminish these risks, we will be evaluating standard measurements of weight and other anthropometric and laboratory tests. Furthermore, to reduce the potential for confounding due to measurement variability, a single investigator will perform all the measurements using the same instruments throughout the study and the same dietitian will perform the nutrition intervention protocol.

### Trial status

The trial is ongoing. Sixty patients have started the study protocol and additional patients are being recruited.

## Additional file

Additional file 1: SPIRIT 2013 checklist: recommended items to address in a clinical trial protocol and related documents. (DOC 122 kb)

## Abbreviations

BMI: Body mass index
FAO: Food and Agriculture Organization
GI: Glycemic index
MDRD: Modification of diet of renal disease
SPIRIT: Standard Protocol Items: Recommendations for Interventional Trials
USDA: United States Department of Agriculture
VAS: Visual analogue scale
